# The Addition of Manganese Porphyrins during Radiation Inhibits Prostate Cancer Growth and Simultaneously Protects Normal Prostate Tissue from Radiation Damage

**DOI:** 10.3390/antiox7010021

**Published:** 2018-01-25

**Authors:** Arpita Chatterjee, Yuxiang Zhu, Qiang Tong, Elizabeth A. Kosmacek, Eliezer Z. Lichter, Rebecca E. Oberley-Deegan

**Affiliations:** 1 Department of Biochemistry and Molecular Biology, University of Nebraska Medical Center, Omaha, NE 68198, USA; arpita.chatterjee@unmc.edu (A.C.); yuxiang.zhu@unmc.edu (Y.Z.); qiangtong@hust.edu.cn (Q.T.); elizabeth.kosmacek@unmc.edu (E.A.K.); Eliezer.lichter@unmc.edu (E.Z.L.); 2 Department of Gastrointestinal Surgery, Union Hospital, Tongji Medical College, Huazhong University of Science and Technology, Wuhan 430022, China

**Keywords:** prostate cancer, manganese porphyrin, radiation, ROS, lipid oxidation

## Abstract

Radiation therapy is commonly used for prostate cancer treatment; however, normal tissues can be damaged from the reactive oxygen species (ROS) produced by radiation. In separate reports, we and others have shown that manganese porphyrins (MnPs), ROS scavengers, protect normal cells from radiation-induced damage but inhibit prostate cancer cell growth. However, there have been no studies demonstrating that MnPs protect normal tissues, while inhibiting tumor growth in the same model. LNCaP or PC3 cells were orthotopically implanted into athymic mice and treated with radiation (2 Gy, for 5 consecutive days) in the presence or absence of MnPs. With radiation, MnPs enhanced overall life expectancy and significantly decreased the average tumor volume, as compared to the radiated alone group. MnPs enhanced lipid oxidation in tumor cells but reduced oxidative damage to normal prostate tissue adjacent to the prostate tumor in combination with radiation. Mechanistically, MnPs behave as pro-oxidants or antioxidants depending on the level of oxidative stress inside the treated cell. We found that MnPs act as pro-oxidants in prostate cancer cells, while in normal cells and tissues the MnPs act as antioxidants. For the first time, in the same in vivo model, this study reveals that MnPs enhance the tumoricidal effect of radiation and reduce oxidative damage to normal prostate tissue adjacent to the prostate tumor in the presence of radiation. This study suggests that MnPs are effective radio-protectors for radiation-mediated prostate cancer treatment.

## 1. Introduction

One of the most common treatments for prostate cancer is radiation therapy. Increased production of reactive oxygen species (ROS) is reported during and after radiation therapy [1]. Radiation-mediated ROS, specifically hydroxyl radicals, cause cytotoxic effects in tumor cells, which inhibits tumor growth [2]. However, some tumor cells escape this initial ROS-mediated cell death and become adapted to the elevated ROS levels, which can cause radio-resistance [3,4]. During radiation, normal tissue adjacent to the tumor, can be damaged directly by radiation or indirectly through bystander effects, which can result in fibrosis and loss of tissue function over time. These events collectively lead to side effects that reduce the quality of life of prostate cancer patients [5,6].

Radiation-mediated side effects are caused by the ongoing production of free radicals in non-targeted normal tissues [7,8]. The majority of free radicals initially arise from the lysis of water molecules. However, free radicals and ROS are released hours to days after radiation. These sources are likely from NADPH oxidases and damaged mitochondria. The acute and chronic elevation of ROS results in damage of DNA, protein and lipids. One such reaction is the non-enzymatic peroxidation of polyunsaturated fatty acids catalyzed by free radicals. Lipid peroxidation is a self propagating chain reaction and the initial oxidation of only a few lipids can result in damage to lipid bilayers, ultimately destabilizing functional and protective membranes, which can cause severe tissue damage. The chemical by-product of this lipid peroxidation is 4-hydroxynonenal (4-HNE), which forms adducts to the histidine, cysteine and lysine residues of proteins, amino group containing lipids and to guanosine moieties of DNA [9]. Therefore, under oxidative stress, such as radiation, the excess load of free radicals leads to lipid peroxidation as measured by the production of 4-HNE.

**Figure 1:**
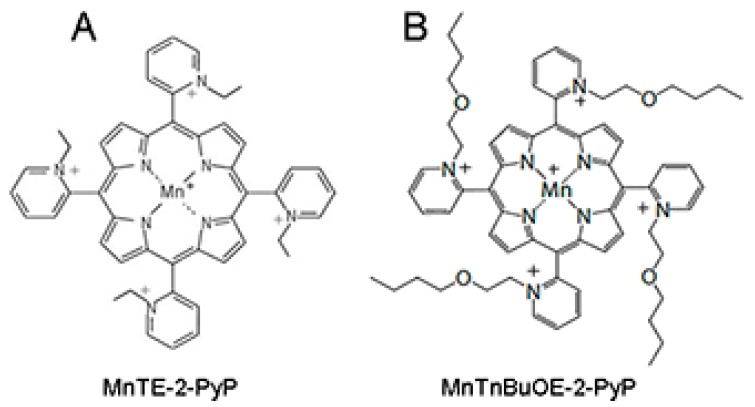
Structure of the two manganese porphyrins, (**A**) MnTE-2-PyP and (**B**) MnTnBuOE-2-PyP. These two porphyrins mimic the active site of native SOD enzymes.

Given the pivotal role of ROS in inducing and propagating normal tissue damage following radiotherapy, a ROS scavenger could be an effective radioprotector. The use of a radioprotector, which can prevent tumor adjacent normal tissue damage during radiation therapy without hindering the radiation-mediated inhibition of tumor growth, would be beneficial for prostate cancer patients undergoing radiation therapy. We have previously described that manganese porphyrins (MnPs: MnTE-2-PyP or MnTnBuOE-2-PyP, Figure 1A,B), which are ROS scavengers, can inhibit the growth of either prostate or colon cancer cells in combination with radiation or chemotherapeutic agents in vitro [10,11]. We have also reported that MnPs protect prostate and colon fibroblasts from radiation-induced damage in vitro [10,12]. We have previously shown that, MnTE-2-PyP protects from radiation damage to normal urogenital tissues in rats [13]. Others have shown that MnTE-2-PyP does not protect prostate cancer cells from radiation killing in hind flank tumor models [14]. However, no one has examined the ability of MnPs in combination with radiation to inhibit tumor growth, while simultaneously protecting normal tissues from radiation damage in a prostate cancer model.

An intriguing question in the field has been, “How do MnPs have different effects on cancer vs. normal cells?” Batinic-Haberle et al. have proposed that MnPs act differently in a normal cell as compared to a tumor cell due to different redox environments of the cells [15]. If the affected cell has inadequate antioxidant defenses, the manganese metal at the active site of the porphyrin may become oxidized and, in turn, act as an oxidizing agent rather than a reducing agent in this environment. Thus, a more oxidizing environment will likely cause the MnPs to act as pro-oxidants and a more balanced redox environment, will cause the MnPs to behave as mild antioxidants. We have previously shown that in normal prostate fibroblasts, MnTE-2-PyP scavenges superoxide and reduces overall ROS in these irradiated cells [12]. Others have shown that when cancer cells are treated with MnPs in combination with ascorbate or other chemotherapeutic drugs, the cancer cells become more oxidatively stressed and results in cell death [16,17,18,19]. However, no one has characterized the effects of these MnPs alone in cells with different basal redox environments.

In the initial disease stage, prostate cancer is dependent on androgens for tumor growth and the tumor growth can be reduced by the removal of androgens [20,21]. As the disease progresses, the tumors become androgen independent and more oxidatively stressed [22]. Therefore, the survival strategies in initial vs. late stage tumor cells are different. We have reported that MnTE-2-PyP can inhibit the growth of both androgen dependent (LNCaP) and independent (PC3) cell lines in the presence of radiation in vitro [11]. Therefore, MnPs could potentially be used in combination with radiation in both androgen dependent and androgen independent tumor models.

To test this hypothesis, we have orthotopically implanted human prostate cancer cell lines (LNCaP or PC3) into the prostates of athymic nude mice. After tumor development, mice were irradiated with X-rays in the presence or absence of MnPs. MnP treatment continued until animals were sacrificed or died. We found that MnP treatment, when combined with radiation, significantly enhanced the antitumor effect of radiation and promoted overall life span of mice in both prostate tumor models. We also measured the ability of MnPs to mitigate radiation-generated 4-HNE levels in the tumor and normal tissue regions that are adjacent to the tumor in both orthotopic cancer models. In both tumor types, we observed increased 4-HNE levels when MnPs were combined with radiation. In contrast, in normal prostate glandular regions neighboring the tumor tissues, we found that both MnPs reduced 4-HNE formation as compared to the irradiated alone animals. These results indicate that the addition of MnPs can maintain normal redox homeostasis in the normal tissue adjacent to the tumor and protect it from radiation damage, while enhancing oxidative stress in the tumors when combined with radiation.

In this study, we also investigated the role of MnPs on superoxide levels and overall redox environment in the prostate cancer cells in vitro. We found that the MnPs behave quite differently in the two prostate cancer cells. In the less aggressive LNCaP cells, we found that both MnPs do not increase hydrogen peroxide levels. In contrast, in PC3 cells, which are highly aggressive, the addition of the MnP resulted in a significant increase in hydrogen peroxide levels, which resulted in the oxidation of protein thiols. We found that LNCaP cells have twice as much catalase activity as PC3 cells, and we postulate that peroxide removal may be a key factor in regulating the activity of the MnPs.

## 2. Materials and Methods

### 2.1. Cell Lines

PC3 and LNCaP cells were purchased from American Type Culture Collection (ATCC, Manassas, VA, USA). Constitutive luciferase expressing PC3 cells (PC3-Luc) were purchased from Applied Biological Materials Inc. (ABM, Richmond, BC, Canada).

To generate constitutive luciferase expressing LNCaP cells (LNCaP-Luc), 50 μL of pre-made lentiviral expression plasmid for firefly luciferase (LVP020-PBS, Amsbio, Cambridge, MA, USA) was transfected into LNCaP cells at 50% confluency. After 72 h of transfection, transfected cells were selectively cultured for two weeks in puromycin (0.4 μg/mL) containing media. Constitutive expression of luciferase was monitored every week using the Tropix LucScreen Assay (Applied Biosystem, Foster City, CA, USA, cat. T2300) and GFP production was observed using a fluorescence microscope (Leica, DM4000 B LED, Buffalo Grove, IL, USA).

Both cell types were cultured in RPMI-1640 media containing, 10% (for PC3) or 5% (for LNCaP) fetal bovine serum (FBS) and 1% penicillin/streptomycin. Cultures were maintained at 37 °C and 5% CO_2_.

### 2.2. Primary Prostate Fibroblast Isolation and Culture Conditions

Prostates were collected from six to eight-week-old C57BL/6 mice [12]. After mincing the prostates, they were digested by 5 mg/mL of collagenase I (Thermo Fisher Scientific, Waltham, MA, USA, cat. 17100017) for 30 min at 37 °C [23]. Tissue fragments were then cultured for 2–3 weeks in Dulbecco’s Minimal Essential Media (DMEM, Hyclone, Logan, UT, USA) supplemented with 10% fetal bovine serum (FBS), 1% penicillin/streptomycin and 1% non-essential amino acids. After 5 days of culture, all the cells were fibroblasts. The purity of the cells were determined by ERTR7 (Santa Cruz, cat. sc-73355), a fibroblast marker and Keratin17 (Cell Signaling cat. 4543) an epithelial cell marker. All experiments were repeated in triplicate using primary fibroblasts cells collected from prostates of different mice.

### 2.3. Animal Husbandry

Six to eight week old, male, athymic mice or C57Bl/6 mice (Charles River Laboratories, Wilmington, MA, USA) were used for experiments. Animals were housed five animals per cage in standard mouse cages in the animal facility at the University of Nebraska Medical Center (UNMC, Omaha, NE, USA) and were exposed to a 12 h light/12 h dark cycle and fed and watered ad libitum. All experimental protocols were reviewed and approved by the UNMC Institutional Animal Care (Omaha, NE, USA) and Use Committee (14-054-08-FC).

### 2.4. Orthotopic Implantation of Tumor Cells

Athymic mice were anesthetized by continuous flow of 2.5% isoflurane with oxygen using a mouse anesthesia machine. A one cm midline incision was made in the lower abdomen after cleaning the skin with an iodine solution. To expose the prostate gland, seminal vesicles and bladder were gently retracted. PC3-Luc or LNCaP-Luc cells (50 μL containing 2.0 × 10^6^ cells in 50% Matrigel) were injected into the dorsal prostatic lobe using a 30-gauge needle. The peritoneal tissues were closed in two layers with absorbable catgut sutures (cat. 563B, Surgical Specialties, Tijuana, Mexico) and the skin was closed with wound clips (cat. 1111C15, Thomas Scientific, Swedesboro, NJ, USA). Buprenorphine (0.1 mg/kg, Reckitt Benckiser Healthcare (UK) Ltd., Hull, UK) was administrated by intraperitoneal route just after the surgery followed by three doses at six, twenty-four and forty-eight h after surgery. Sterile surgical procedures were maintained for the entire process. Wound clips were removed after ten days and animals were monitored for infection or distress.

### 2.5. Bio-Luminescence Imaging

d-Luciferin potassium salt (100 mg/kg, PerkinElmer, cat. 122799, Waltham, MA, USA) was dissolved in sterile PBS and injected intraperitoneally into the tumor bearing mice 15 min prior to imaging. For imaging the luciferase expressing tumors, mice were anesthetized using 2.5% isoflurane with oxygen and placed in the Xenogen IVIS Spectrum bioluminescence imaging system (PerkinElmer, MA, USA). Images were acquired and analyzed by Living Image 4.5.1 software (Caliper Life Sciences, Hopkinton, MA, USA) with an exposure time of one second. Regions of interest (ROI) were determined to encompass the area with the most intense light, and signal intensity was calculated based on a measurement of photons/s/cm^2^/sr.

### 2.6. Radiation and MnP Administration Protocol

Scheme/timeline used for LNCaP or PC3 tumor models:

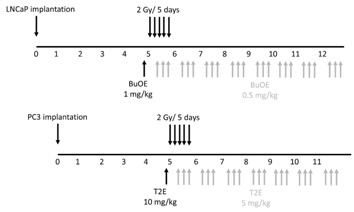


Five weeks after surgery, tumors were imaged as described in the bioluminescence imaging section. Animals bearing very large tumors, no tumors, or very small tumors were excluded from the rest of the study. The average tumor size was 200 mm^3^. Forty mice (10 mice per group) from the PC3-Luc cell implanted group and 32 mice (8 mice per group) from the LNCaP-Luc implanted group were used for further experiments. This ensured that all the tumors were similar size before starting the treatment protocol. The mice were divided into four treatment groups for PC3 and LNCaP models: 1. PBS only 2. MnPs only 3. Radiation only and 4. Radiation + MnPs. Both PC3 and LNCaP tumors were irradiated with X-rays (2 Gy per day, for five sequential days) to their lower abdomen using lead shielding (Rad Source RS-2000 Biological System). A single dose of PBS (for controls) or 1 mg/kg MnTnBuOE-2-PyP (BuOE) for LNCaP bearing mice, or 10 mg/kg MnTE-2-PyP (T2E) for PC3 bearing mice was administrated 24 h prior to the first radiation dose. MnTE-2-PyP and MnTnBuOE-2-PyP were kind gifts from Dr. James Crapo (National Jewish Health, Denver, CO, USA). During and after irradiation for both tumor types, half of the mice from each of irradiated and non-irradiated groups were treated with MnPs intraperitoneally, three times a week. The maintenance dosing was half the pre-radiation loading doses reported above. The control mice from each of radiated and non-radiated groups were treated with an equal volume of PBS, three times a week.

### 2.7. Tumor Harvesting, Tumor Size Measurement and Enumerating Metastatic Lesions

From LNCaP-Luc implanted mice, three mice from each of the four groups were sacrificed and the tumors were harvested three weeks post-irradiation. In the case of PC3-Luc implantation, five mice from each of the four groups were sacrificed and the tumors were harvested two weeks post-radiation. PC3 cells grow more quickly than LNCaP cells, which is why LNCaP cells were allowed to grow an additional week after implantation. The width and length of the excised tumor was measured with calipers and the volume was estimated according to the formula: [(width)^2^ × length/2]. After measurement of the primary tumor, half of the tumor was flash frozen and stored at −80 °C. The other half was fixed in 4% formalin followed by 70% ethanol. These tissues were paraffin embedded and 7 μm sections were cut and placed on slides for immunostaining by the Tissue Science Facility at the University of Nebraska Medical Center (Omaha, NE, USA).

The number of metastatic nodules at the distal sites were enumerated for every animal by visual inspection and verified as true metastases using the LucScreen assay. The animals were placed in one of four groups: no observable metastases, 1 metastatic nodule, 2 metastatic nodules, and 3 or more metastatic nodules.

### 2.8. Tumor Growth and Overall Animal Survival

The remaining animals were continuously administered MnTnBuOE-2-PyP or MnTE-2-PyP or PBS, three times a week until sacrifice. Animals were imaged every week to monitor tumor size using the Xenogen IVIS Spectrum bioluminescence imaging system after injection of d-Luciferin potassium salt, as described above. The average signal intensity per tumor was calculated for every animal group and plotted over time.

Animals were euthanized when the tumor volume began to influence health status and/or mobility. For survival curve analysis, total life span in days after irradiation was calculated and plotted against percent survival in all groups. Kaplan Meier survival analysis was performed using GraphPad prism 6.0.5 software (La Jolla, CA, USA).

### 2.9. Tumor Lysate Preparations and Western Blot

Frozen tumor samples were minced and homogenized (Pro scientific, Oxford, CT, USA) in lysis buffer [120 mM NaCl, 50 mM Tris-HCl, 5 mM EDTA, 1% NP-40 and complete protease inhibitor cocktail tablets (1 tablet/50 mL, cat. 11697498001; Roche Diagnostics, Indianapolis, IN, USA)]. After incubation on ice for 30 min, the tissue homogenates were sonicated for 8–10 pulses at 80% amplitude. Lysates were then centrifuged for 15 min at 12,000× *g* and protein concentrations of the supernatants were determined by using the Bradford Assay (Amresco, cat. E530). Tumor tissue lysate (40 μg) was electrophoresed on a 4–12% gel, transferred to a nitrocellulose membrane, and blocked in 5% milk for two hours. To examine PCNA expression, membranes were incubated overnight at 4 °C with a PCNA antibody (BD Transduction laboratories, cat. 610665, 1:1000 dilution). Mouse secondary antibodies (1:10,000 dilution, cat. A24524; Invitrogen, Carlsbad, CA, USA) were incubated for one hour and the blots were developed using ECL (cat. 80196, Pierce ECL2 western blotting substrate) and exposed to film. Densitometry was performed on the blots using ImageJ analysis software 1.50i (National Institutes of Health, Bethesda, MD, USA).

### 2.10. Immunohistochemistry

Fixed tumor tissue that also contained normal prostate tissue, was paraffin embedded and sectioned by the Tissue Science Facility at the University of Nebraska Medical Center. Sections were then immunostained for a marker of oxidative stress, 4-hydroxynonenal (4-HNE). Tissues were de-paraffinized in xylenes and rehydrated through graded alcohols. For antigen retrieval, slides were heated to 95 °C in 0.01 M sodium citrate buffer (pH 6.0) with 0.05% Tween 20. Slides were then allowed to cool in phosphate buffer (pH 7.0) for 30 min. For blocking, 4-HNE staining required the use of a M.O.M. ™ kit (Vector Labs, Burlingame, CA, USA, cat. BMK-2202) and was used according to the manufacturer’s directions. Following blocking, tissue sections were incubated with a primary antibody (4-HNE, 1:50, R&D Systems, Minneapolis, MN, USA, cat. MAB3249) overnight at 4 °C in a humidified chamber. The following day, slides were washed in Super Sensitive Wash Buffer (BioGenex Laboratories, Fremont, CA, USA, cat. HK583-5K) and stained with a secondary antibody conjugated to AlexaFluor555 (1:100, goat anti-mouse, Invitrogen, cat. A21422). Slides were mounted under coverslips with ProLong™ Gold Antifade with DAPI (Invitrogen, cat. P36931). Areas of normal prostate glandular tissue were imaged using a Leica DM 4000B LED fluorescent microscope, followed by analysis with ImageJ. The normal prostate glandular region and the tumor tissue were selected for further analysis. The epithelial cell layer, omitting the glandular lumen area, was manually traced and the intensity of 4-HNE staining was measured. Tumor tissues were manually traced and the intensity of 4-HNE staining was measured. Average raw integrated intensity per unit area was calculated. Statistical significance was determined using 1-way ANOVA followed by post hoc Tukey’s multiple correction test.

### 2.11. Measurement of Superoxide

To measure the superoxide production, PC3 or LNCaP cells were seeded at a concentration of 0.5 × 10^6^ cells/flask in the presence or absence of MnTE-2-PyP (30 μM) or MnTnBuOE-2-PyP (0.5 μM) or an equal volume of PBS overnight. In some cases, cells were irradiated with 2 Gy of X-rays, then harvested by trypsinization 48 h post-radiation. After washing, cells were stained with dihydroethidium (DHE, 5 μM) for 20 min at 37 °C in the dark and then subjected to flow cytometric analysis using a LSRII Green 532 Flow Cytometer (BD Biosciences, San Jose, CA, USA). In order to measure superoxide specifically, 405/570 nm excitation/emission was used. Data was analyzed using FACSDiVa analysis software v8.0.2 (BD Biosciences, San Jose, CA, USA).

### 2.12. Detection of Intracellular Hydrogen Peroxide Levels

PC3 or LNCaP cells were seeded in chamber slides (ThermoFisher Scientific, cat. 05031780) and treated with PBS, MnTnBuOE-2-PyP (0.5 μM), or MnTE-2-PyP (30 μM) overnight. In some cases, cells were irradiated with 2 Gy of X-rays 24 h before hydrogen peroxide staining began. Cells were then treated with DMSO or Peroxy Orange 1 (15 μM, Fisher Scientific, cat. 4944) for one hour in the dark at 37 °C. The fluorescence was detected by a Leica DM 4000B LED fluorescent microscope with the Ex/Em at 555 nm/565 nm. An average of five images were taken for each condition. The average intensity per cell was calculated based on a minimum number of 100 cells for each condition, and analysis was performed using ImageJ.

### 2.13. Detection of Thiol Oxidation

The method of detecting thiol oxidation levels has been described previously [24]. Briefly, cells were treated with PBS, MnTnBuOE-2-PyP (0.5 μM), or MnTE-2-PyP (30 μM) for three days. In some cases, 24 h before harvest, the cells were exposed to 2 Gy of irradiation with or without MnP treatment. Cells were homogenized in lysis buffer (120 mM NaCl, 50 mM Tris-HCl, 5 mM EDTA, 1% NP-40 and complete protease inhibitor cocktail) and incubated for 10 min on ice. Lysates were centrifuged at 4 °C for 7 min at 12,000× *g*, and the supernatants were collected. Protein concentrations were measured by the Bradford assay. The final concentration of each sample was adjusted to 1 mg/mL. Lysates were then treated with distilled water, or 1 mM DTT, or 1 mM diamide at room temperature for 30 min. To isolate proteins with reduced thiols, protein lysate (20 μg) was mixed with 970 μL of binding buffer (0.1% SDS in PBS) and 5 μL of 30 μM *N*-(Biotinoyl)-*N*′-(Iodoacetyl)Ethylenediamine (BIAM) (Invitrogen, Carlsbad, CA, USA, cat. B1591). The whole reaction system was incubated for 30 min at room temperature in the dark. The reaction was terminated by the addition of 50 μL of 500 mM β-mercaptoethanol. Streptavidin-agarose beads (100 μL) (ThermoFisher Scientific, cat. SE243295) were then mixed with samples for one hour at room temperature. The streptavidin-agarose-bound complex was washed four times by adding 1 mL of binding buffer and centrifuged at 850× *g*. Samples were boiled at 75 °C for 10 min with 4 μL of 10× reducing agent, 10 μL 4× loading dye, and 26 μL distilled water. Samples were run on the Bolt^TM^ 4–12% Bis-Tris Plus gels (Invitrogen, cat. NW04120BOX) and transferred to the nitrocellulose membrane (Invitrogen, cat. IB23001). The western blots were probed with an streptavidin-HRP antibody (1:10,000) (ThermoFisher Scientific, cat. QG223359) and bands were detected with ECL reagent.

### 2.14. Catalase Activity Staining Assay

Catalase activity staining assay was performed as described before [24]. Briefly, cells were treated with PBS or MnTE-2-PyP (30 μM) or MnTnBuOE-2-PyP (0.5 μM) overnight. In some cases, cells were irradiated with 2 Gy and harvested 24 h later. Cells were homogenized in lysis buffer (120 mM NaCl, 50 mM Tris-HCl, 5 mM EDTA, 1% NP-40 and complete protease inhibitor cocktail) and incubated for 10 min on ice. Lysates were centrifuged at 4 °C for 7 min at 12,000× *g*, and the supernatants were collected. Protein concentrations were measured by the Bradford assay. To measure catalase activity, proteins (30 μg) were loaded onto the 10% Mini-PROTEAN TGX precast gel (Bio-Rad, Hercules, CA, USA, cat. 4561033) and run at 100 V for 2 h at 4 °C. The gel was extensively rinsed with distilled water three times, for 10 min each and then soaked in 0.003% H_2_O_2_ for 10 min. The staining solution (2% ferric chloride and 2% potassium ferricyanide in distilled water) was poured onto the gel. The achromatic bands are indicative of catalase activity. Gel images were inverted and densitometry of the bands were performed using Image J.

### 2.15. Statistical Analysis

GraphPad Prism 6.0.5 was used for all the statistical analyses. Mean and standard deviation values from three independent experiments were used for statistical analysis for all the experiments performed. Unless otherwise described, significant differences between the groups was determined by a 1-way ANOVA test followed by a post hoc Tukey’s test for multiple comparisons or a student’s *t*-test. For the non-invasive tumor size measurement, linear regression modeling was used to determine differences between slopes. For survival analysis, a Kaplan Meier curve was plotted. The percent survival and median survival in days were documented and compared between groups by using the Mantel-Cox test.

## 3. Results

### 3.1. MnTnBuOE-2-PyP Inhibits Growth of PC3 and LNCaP Cells In Vitro

**Figure 2:**
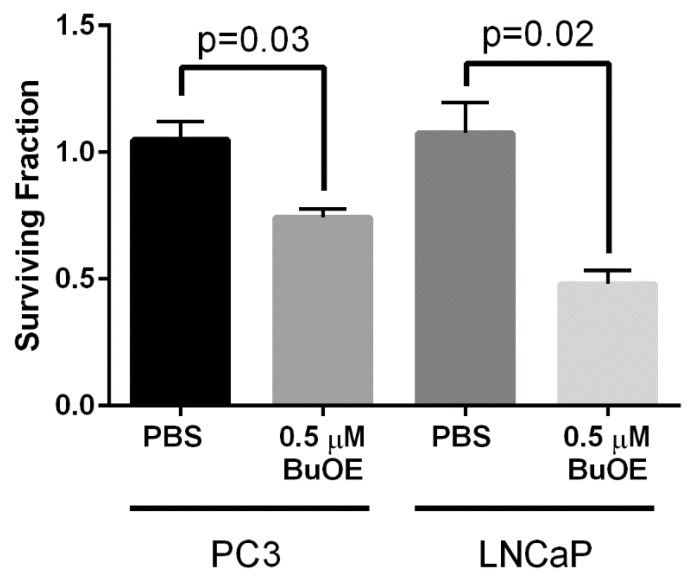
MnTnBuOE-2-PyP inhibits colony formation of prostate cancer cells. Clonogenic assays were performed on PC3 or LNCaP cells with or without MnTnBuOE-2-PyP (0.5 μM). The addition of MnTnBuOE-2-PyP significantly inhibited growth of both prostate cancer cell lines. Data represent mean ± SD from three independent experiments.

Our laboratory has previously shown that MnTE-2-PyP (30 μM) inhibits colony formation of both PC3 and LNCaP cells by ~50% in vitro [11]. We wanted to determine whether MnTnBuOE-2-PyP had similar effects on prostate cancer growth. We found that MnTnBuOE-2-PyP (0.5 μM) significantly inhibited PC3 colony growth by 30% and LNCaP growth by 50% (Figure 2). Thus, MnTE-2-PyP and MnTnBuOE-2-PyP have similar inhibitory effects on prostate cancer growth in vitro.

### 3.2. MnPs Decrease Primary Tumor Volume

We next wanted to determine the effects of these drugs on prostate cancer in vivo and in combination with radiation. MnTnBuOE-2-PyP is in phase I clinical trials for glioblastoma and head and neck cancer patients as a radioprotector. The most common prostate cancer diagnosed in human patients are androgen sensitive tumors [25]. These tumor types are routinely treated with radiation, so we investigated the effects of MnTnBuOE-2-PyP in an orthotopic model of androgen sensitive prostate cancer, the LNCaP cancer model. Since most of the published work on MnPs and prostate cancer has been done with MnTE-2-PyP, we also investigated the effect of MnTE-2-PyP in a more aggressive PC3 prostate cancer model. MnTnBuOE-2-PyP is much more lipophilic than MnTE-2-PyP but they have essentially the same rate constant for dismuting superoxide to hydrogen peroxide [26]. Thus, MnTnBuOE-2-PyP is needed at about 1/10 the dose of MnTE-2-PyP to observe similar changes in cells and animals [26]. Therefore, the dosing of both cells and animals with MnTE-2-PyP is about 10 times more than what is used for MnTnBuOE-2-PyP for the experiments performed in this current study.

**Figure 3:**
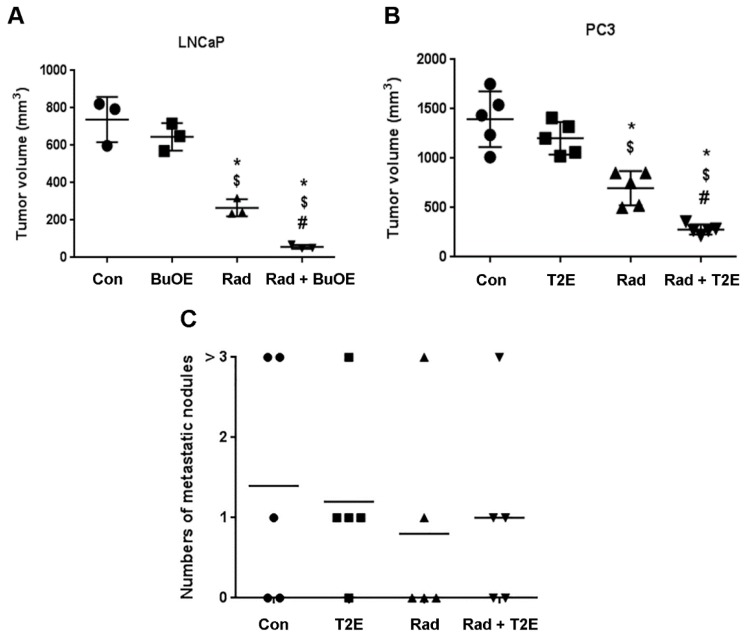
MnPs decrease primary tumor volume. (**A**) LNCaP (**B**) or PC3 cells were orthotopically implanted into mouse prostates. Five weeks post-implantation, the lower abdominal area of mice in the RAD groups, were irradiated with X-rays (2 Gy/day for 5 days). After irradiation, PBS or MnTnBuOE-2-PyP (BuOE, 0.5 mg/kg) (**A**) or MnTE-2-PyP (T2E, 5 mg/kg) (**B**) was injected three times a week. After three (**A**) or two (**B**) weeks of treatment, tumors were harvested and the volumes were calculated. (**C**) After sacrificing the mice, the number of metastatic nodules in the peritoneal cavity were measured and documented visually in PC3 tumor bearing mice. Significance among tumor sizes were determined using a 1-way ANOVA followed by the post hoc Tukey’s for multiple comparisons test. The symbol (*) denotes a significant difference as compared to control group; the symbol ($) denotes a significant difference as compared to the BuOE or T2E group; and the symbol (#) denotes a significant difference when compared to the RAD group, (*n* = 3 for all four groups in the LNCaP model and *n* = 5 for all the groups in the PC3 model).

Five weeks following orthotopic implantation of LNCaP or PC3 cells into the mouse prostates, the lower abdominal area of the mice in the RAD groups, were irradiated with X-rays (2 Gy/day, for 5 consecutive days). After irradiation, either PBS or MnP (MnTnBuOE-2-PyP or MnTE-2-PyP) was injected three times a week. After two weeks post-radiation (for PC3) or three weeks post-radiation (for LNCaP), mice (*n* = 3 for LNCaP and *n* = 5 for PC3) were sacrificed and tumors excised. Combined treatment of MnPs with radiation decreased the average tumor volume by 92.3% in LNCaP tumors and 80.0% in PC3 tumors as compared to the PBS treated group (Figure 3A,B). Whereas, radiation alone decreased the average tumor volume by 64.0% and 50.0% in the respective tumor models. Therefore, the addition of MnPs significantly enhanced the anti-tumor activity of radiation, by 28.25% (in the LNCaP model) and 29.96% (in the PC3 model) (Figure 3A,B). MnPs significantly decreased tumor volumes in the presence of radiation in both of the prostate cancer models at this early time point.

### 3.3. MnPs Do Not Increase Metastasis

Metastatic prostate cancer is the major cause of prostate cancer related deaths. The American Cancer Society reported in 2017, the five-year survival rate of prostate cancer patients with distant metastasis is only 28%. Therefore, it was necessary to examine the effect of MnPs on metastasis. During the tumor harvest two weeks (for PC3) or three weeks (for LNCaP) after irradiation, the number of metastatic nodules was documented. In the LNCaP tumor model, no metastatic nodules were observed in any of the groups (data not shown). In contrast, for the PC3 model, metastatic nodules were observed in the inguinal lymph nodes, liver, peritoneal cavity and upper digestive tract. We found no significant differences in the numbers of metastatic nodules among any of the groups (Figure 3C). This study suggests that MnPs do not affect metastatic progression of the PC3 tumors.

### 3.4. MnPs Decrease Primary Tumor Growth Over Time

**Figure 4:**
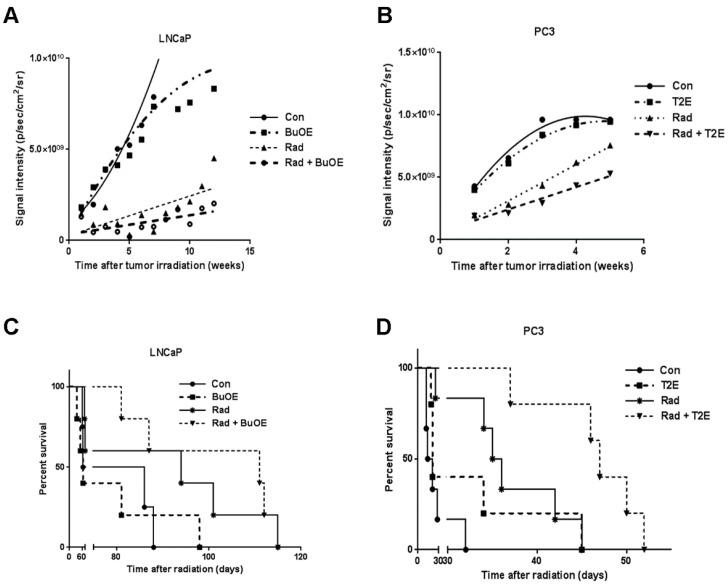
MnPs decrease the tumor growth rate and increases survival of tumor bearing mice. LNCaP or PC3 cells were orthotopically implanted into mouse prostates. Five weeks after implantation, the lower abdominal area of mice in the RAD groups, were irradiated with X-rays (2 Gy/day for 5 days). After irradiation, PBS or MnTnBuOE-2-PyP (BuOE, 0.5 mg/kg) or MnTE-2-PyP (T2E, 5 mg/kg) was injected three times a week during the life span of the mouse. Tumor sizes were measured once a week by non-invasive imaging. (**A**) LNCaP tumor growth; (**B**) PC3 tumor growth. All the data points were statistically analyzed by linear regression modeling. Tumor growth rate in the LNCaP model was significantly reduced in RAD + BuOE group as compared to the RAD only group (*p* value = 0.008). In the PC3 model, tumor growth rate was significantly reduced in the RAD + T2E group as compared to the RAD only group (*p* value = 0.013). Radiation significantly reduced the tumor growth rate in both models; (**C**) Survival curve for mice bearing LNCaP tumors; (**D**) Survival curve for mice bearing PC3 tumors. Kaplan-Meier survival curves were plotted and median life span in days after therapy was calculated in all groups. Differences between median survival was determined by the Log-rank (Mantel-Cox) test. In both tumor models, radiation increased the median survival as compared to controls and RAD + MnP treatment further increased the median survival as compared to the RAD only group. In the LNCaP model, these increases were not statistically significant but in the PC3 model, these increases were significant. LNCaP model: *n* = 4 for PBS only group, *n* = 5 for all other groups. PC3 model: *n* = 6 for PBS and RAD only groups, *n* = 5 for T2E and RAD + T2E groups.

Luciferase expressing tumor cells were tracked by injecting the tumor bearing animals with luciferin followed by bio-imaging once a week after irradiation throughout the life span of the mouse. The luminescence signal reflects the number of luciferase expressing cancer cells, which corresponds to tumor size. The average luminescence signal intensity for each group was plotted against time. Using linear regression modeling, the curves were fit to the data and the slopes of the curves were analyzed (Figure 4A,B). Not surprisingly, we found that in both models, radiation decreased tumor growth when compared to the PBS control group. Tumor growth was further significantly inhibited in the groups treated with radiation in the presence of MnPs, (*p* value = 0.008 in LNCaP and *p* value = 0.013 in PC3) as compared to the irradiated alone groups (Figure 4A,B). This study revealed that MnPs treatment further inhibited prostate tumor growth in combination with radiation.

### 3.5. MnPs in Combination with Radiation Increases Median Survival

To determine the effect of MnPs on the survival of prostate cancer tumor bearing mice, we have generated Kaplan-Meier survival curves for each treatment group (Figure 4C,D). In the LNCaP model, median survival was 64, 75, 94, and 111 days in control, MnTnBuOE-2-PyP, radiation, and MnTnBuOE-2-PyP combined with radiation groups, respectively (Figure 4C). In the PC3 model, control, MnTE-2-PyP, radiation, and MnTE-2-PyP combined with radiation groups have median survivals of 17.5, 21, 35.5 and 47 days respectively (Figure 4D). In both tumor models, MnPs increased the survival of the mice in the presence of radiation. Median survival of LNCaP bearing mice was further increased by seventeen days when animals were treated with radiation in combination with MnTnBuOE-2-PyP as compared to irradiated alone mice. However, this increase in median survival was not significantly different between these groups. In the PC3 tumor model, the median survival of irradiated mice was increased significantly by eighteen days as compared to control (*p* value = 0.0030). Median survival of the mice in the irradiated and MnTE-2-PyP treated group was significantly increased by twelve days as compared to the irradiated only group (*p* value = 0.0080). (Figure 4C,D). Thus, our study revealed that MnPs do not interfere with the anti-tumor action of radiation but rather enhance post-radiation median survival in both models of prostate cancer.

### 3.6. MnPs, in Combination with Radiation, Decrease Prostate Cancer Cell Proliferation

**Figure 5:**
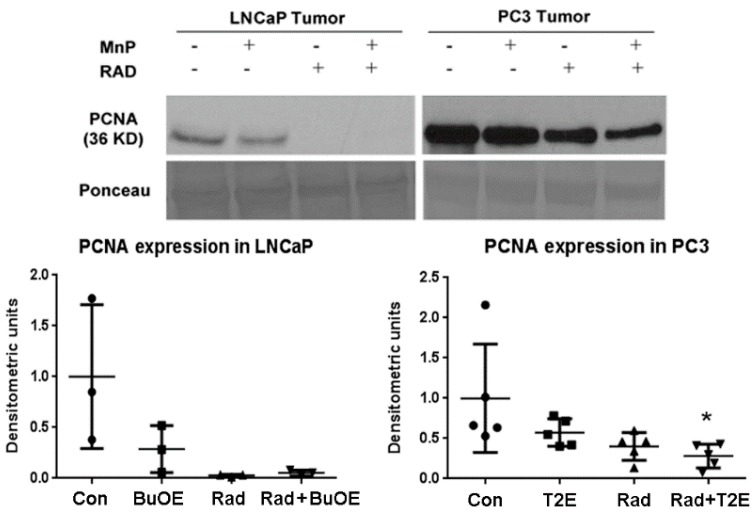
In combination with radiation, MnPs decrease PCNA expression. After harvesting the tumor, 40 μg of tumor lysates (*n* = 3 for LNCaP and *n* = 5 for PC3) were used to measure the expression levels of PCNA by western blot. Ponceau staining was used to confirm equal protein loading. Densitometry was quantified using ImageJ. Significant change in PCNA expression level was calculated by a 1-way ANOVA followed by post hoc Tukey’s multiple correction test. The symbol (*) denotes a significant difference as compared to controls.

As MnPs decrease tumor progression and tumor volume, we tested whether MnPs can affect proliferation of tumor cells by performing western blots for the expression of the proliferation cell nuclear antigen (PCNA) in the tumor lysates obtained 2–3 weeks post-radiation. In LNCaP tumors, expression of PCNA was markedly reduced by radiation, or when radiation was combined with MnTnBuOE-2-PyP as compared to controls, but this decrease was not statistically significant (*p* value = 0.0504 and 0.0566 in the case of RAD only and RAD + BuOE group respectively). This may be partly due to the small sample size (*n* = 3) for each group. In the PC3 tumor model, MnTE-2-PyP, in combination with radiation, significantly decreased PCNA expression (*p* value = 0.0311, Figure 5).

### 3.7. MnPs Enhance Lipid Oxidation in Prostate Cancer Tissues in Combination with Radiation Therapy

**Figure 6:**
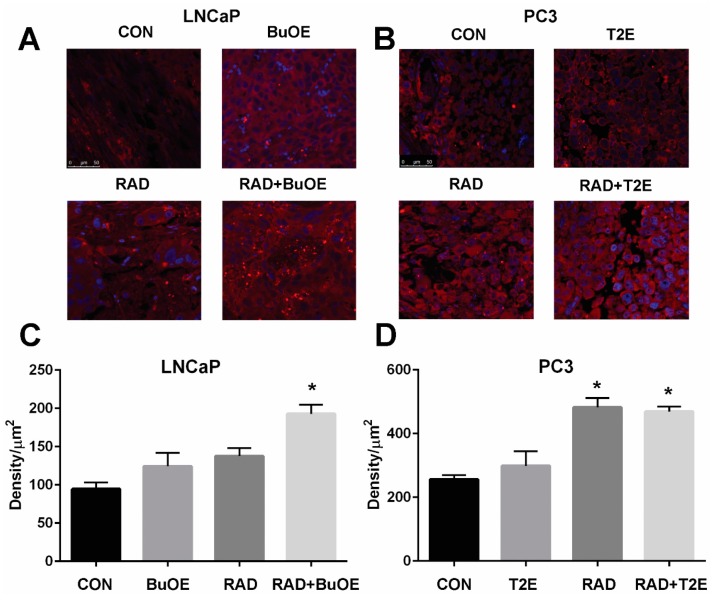
MnPs, in combination with radiation, induce lipid peroxidation in prostate cancer tissues. Formalin fixed paraffin embedded prostate tissue was stained and imaged for 4-HNE. 4-HNE was quantified in the prostate tumor tissues. Images were analyzed using ImageJ software. Average raw integrated intensity per unit area was plotted. The magnification bar in the lower left panels denotes 50 μm. (**A**) Representative images of the four groups for LNCaP tumors are shown in panels; (**B**) Representative images of the four groups for PC3 tumors are shown in panels; (**C**) Quantification of LNCaP images; (**D**) Quantification of PC3 images. The symbol (*) denotes a significant difference as compared to control group. In the LNCaP model, *n* = 3 for all groups. In the PC3 model, *n* = 4 in PBS only and RAD only groups and *n* = 5 in T2E and RAD + T2E groups.

Lipid oxidation, as indicated by 4-HNE staining, was significantly enhanced three weeks after radiation therapy for LNCaP tumors or two weeks after radiation exposure for PC3 tumors from irradiated mice treated with MnPs (Figure 6). Radiation alone resulted in significant lipid peroxidation in PC3 tumors but not in LNCaP tumors, although there was a trend of increased 4-HNE staining in these animals (Figure 6). The addition of either MnTE-2-PyP or MnTnBuOE-2-PyP alone did not significantly affect lipid peroxidation in the tumor tissues, although there was a trend of increased 4-HNE levels (Figure 6). PC3 tumors had higher levels of 4-HNE overall as compared to LNCaP cells, indicating that PC3 cells are more oxidatively stressed than LNCaP cells, even at baseline.

### 3.8. MnPs Reduce Lipid Peroxidation in Normal Prostate Tissues Adjacent to Prostate Cancer after Radiotherapy

**Figure 7:**
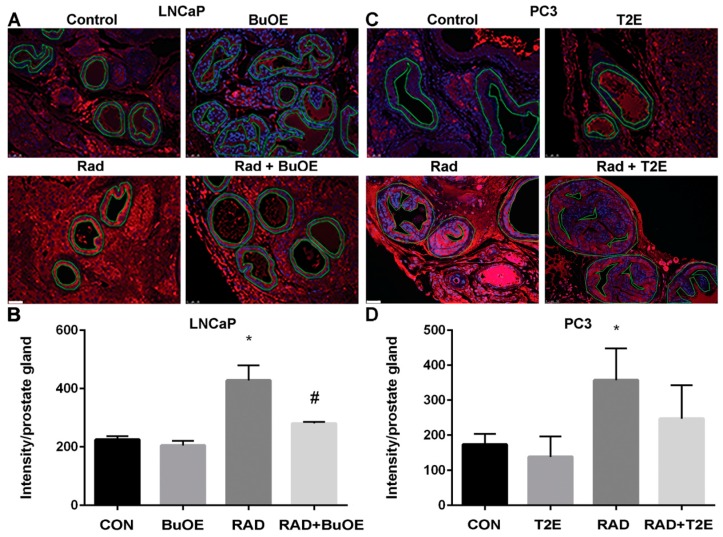
MnPs decrease radiation-induced lipid peroxidation in normal prostate tissues adjacent to the tumor. Formalin fixed paraffin embedded prostate tissue was stained and imaged for 4-HNE. 4-HNE was quantified in the epithelial cells of the normal prostate glands (outlined in images). (**A**) Representative images of the four groups for LNCaP tumors; (**B**) Quantification of LNCaP images; (**C**) Representative images of the four groups for PC3 tumors; (**D**) Quantification of PC3 images. Images were analyzed using ImageJ software. Average raw integrated intensity per unit area was plotted. The magnification bar in the lower left panels denotes 50 μm. The symbol (*) denotes a significant difference as compared to control group and the symbol (#) denotes a significant difference when compared to the RAD group. In the LNCaP model, *n* = 3 for all groups. In the PC3 model, *n* = 4 in PBS only and RAD only groups and *n* = 5 in T2E and RAD + T2E groups.

Radiation causes oxidative damage to normal tissues adjacent to the tumor [27]. Lipid peroxidation, a well-known marker of oxidative damage, was measured by the production of 4-HNE. To further demonstrate that MnPs are radioprotectors, we quantified the levels of 4-HNE in the normal prostate glandular tissues adjacent to the tumor from both prostate cancer models. Radiation caused a significant increase in 4-HNE levels in the prostate glands of both LNCaP and PC3 tumor models as compared to controls (Figure 7). In the LNCaP tumor model, radiation significantly increased 4-HNE levels in normal tissues as compared to controls (*p* value < 0.0001). MnTnBuOE-2-PyP treatment significantly decreased 4-HNE levels in normal tissues in irradiated mice as compared to the RAD only group (*p* value = 0.0009, Figure 7). In the PC3 tumor model, radiation significantly increased 4-HNE levels in normal tissues as compared to controls (*p* value = 0.0318). MnTE-2-PyP reduced 4-HNE levels, but the differences were not statistically significant as compared to irradiated alone animals.

### 3.9. MnPs Affect Superoxide Levels Differently in PC3 Cells as Compared to LNCaP Cells

**Figure 8:**
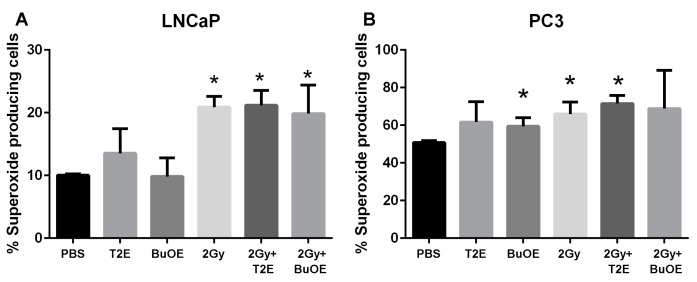
MnPs do not scavenge superoxide in prostate cancer cells. PC3 or LNCaP cells treated with MnTE-2-PyP (30 μM), or MnTnBuOE-2-PyP (0.5 μM) or an equal volume of PBS. In some cases, cells were irradiated (2 Gy) in the presence or absence of the respective MnPs. Cells were stained with DHE and subjected to flow cytometric analysis. (**A**) Percent superoxide producing cells (405 nm) in LNCaP cells; (**B**) Percent superoxide producing cells (405 nm) in PC3 cells. The data are representative of three independent experiments and are presented as the mean ± the standard deviation. The (*) symbol denotes significant difference compared to the PBS group for each cell line. Significance was determined using 1-way ANOVA followed by a student *t*-test.

MnPs are superoxide scavengers, but in certain cell types they can also act as pro-oxidants [26]. In our animal model, the MnP are acting as antioxidants by reducing 4-HNE levels in normal tissues (Figure 7), but enhancing 4-HNE in tumor tissues (Figure 6). To determine what role MnPs were playing in the prostate cancer redox state, we measured DHE activation. Excitation at 405 nm can specifically measure the by-product of DHE and superoxide [12]. We have previously published that MnTE-2-PyP significantly inhibits superoxide in normal primary prostate fibroblast by 2-fold [12]. In contrast to normal cells, MnP treatment did not result in superoxide scavenging (Figure 8). In fact, there was a trend of both MnP treatment causing a small increase in superoxide levels in the cancer cells, but this was not significant (Figure 8). Radiation (2 Gy) resulted in a significant elevation of superoxide levels in both LNCaP and PC3 cells; however, this overall increase was more pronounced in the LNCaP cells. The addition of the MnP with radiation had no effect on these increased levels of superoxide (Figure 8). PC3 cells had a significant higher percentage of cells producing superoxide at baseline as compared to LNCaP cells, again indicating that PC3 cells are more oxidatively stressed than LNCaP cells.

### 3.10. MnPs Enhance Hydrogen Peroxide Levels in PC3 Cells But Not LNCaP Cells

**Figure 9:**
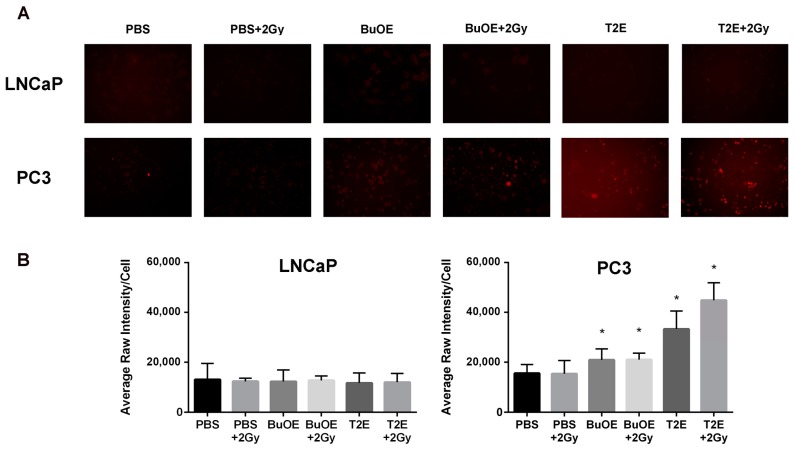
MnTE-2-PyP and MnTnBuOE-2-PyP significantly enhance intracellular hydrogen peroxide in PC3 cells but not in LNCaP cells. (**A**) Representative images of LNCaP (top panel) and PC3 (bottom panel) cells treated with either MnTE-2-PyP (30 μM), MnTnBuOE-2-PyP (0.5 μM) or PBS ± radiation (2 Gy) and stained with peroxy orange 1 (PO1), a probe that fluoresces in the presence of hydrogen peroxide; (**B**) Quantification of the PO1 staining in LNCaP and PC3 cells treated with either MnTE-2-PyP, MnTnBuOE-2-PyP or PBS ± radiation (2 Gy). The symbol (*) denotes a significant difference as compared to control group (PBS for each respective cell line). Data represent mean ± SD from three independent experiments.

Since MnPs were not scavenging superoxide or the MnPs are not reducing superoxide as quickly as it is being produced in the cancer cells, we wanted to investigate the levels of hydrogen peroxide in the cancer cells with MnP treatment. We found that treatment with MnTnBuOE-2-PyP or MnTE-2-PyP significantly increased hydrogen peroxide levels in PC3 cells (Figure 9). In contrast, treatment with the MnPs did not enhance intracellular hydrogen peroxide levels in LNCaP cells (Figure 9). Radiation alone or in combination with MnP had little effect on hydrogen peroxide levels in both cell lines. However, in PC3 cells, radiation in combination with MnTnBuOE-2-PyP or MnTE-2-PyP resulted in a significant enhancement of hydrogen peroxide levels as compared to untreated PC3 cells (Figure 9). In normal prostate fibroblasts, MnP treatment did not result in increased hydrogen peroxide levels either (data not shown).

### 3.11. MnPs Enhance Thiol Oxidation in PC3 Cells But Not LNCaP Cells

**Figure 10:**
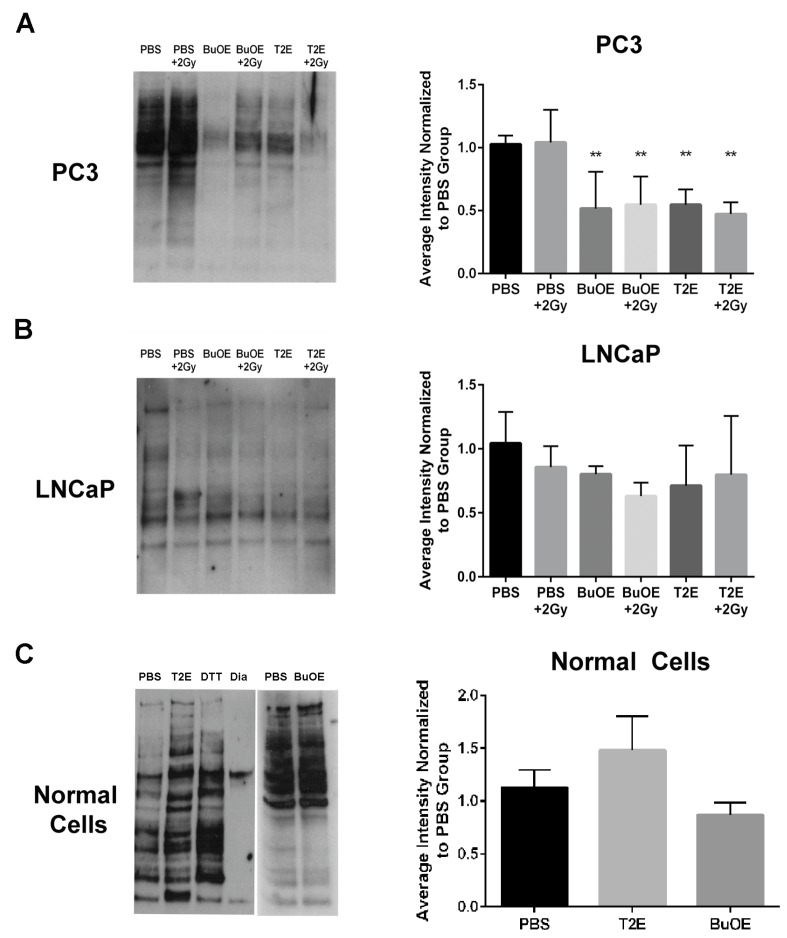
MnTE-2-PyP and MnTnBuOE-2-PyP enhance thiol oxidation in PC3 cells but not in LNCaP cells or normal cells. (Left Panels) Representative images of total reduced thiols by binding to BIAM beads from whole cell lysates treated with MnTE-2-PyP, MnTnBuOE-2-PyP or PBS ± radiation. (Right Panels) (**A**) LNCaP cells. (**B**) PC3 cells. (**C**) Normal cells. Densitometric analysis was performed on three separate blots as shown to the right of each blot. As a control, the lysates were treated with the reducing agent dithiothreiotol (DTT) or the oxidizing agent diamide (Dia). MnTE-2-PyP and MnTnBuOE-2-PyP enhance thiol oxidation in PC3 cells ± radiation but not in LNCaP cells or normal prostate fibroblasts. The symbol (*) denotes a significant difference as compared to control group (PBS for each respective cell line). Data represent mean ± SD from three independent experiments.

Hydrogen peroxide can directly oxidize thiol containing amino acids. Therefore, we wanted to determine if these increased hydrogen peroxide levels had an effect on thiol oxidation of intracellular proteins in PC3 cells. Using a *N*-(Biotinoyl)-*N*′-(Iodoacetyl)Ethylenediamine (BIAM) assay, where the BIAM binds only to reduced thiols, we found that MnTE-2-PyP or MnTnBuOE-2-PyP treated PC3 cells had significantly fewer reduced thiols present in the cell lysate as compared to untreated cells (Figure 10A). This indicates that MnP treatment results in oxidation of thiols in PC3 cells. Radiation (2 Gy) had little effect on thiol oxidation. In accordance with the hydrogen peroxide data, MnPs did not cause oxidation of thiols in LNCaP cells (Figure 10B). The addition of MnPs to normal primary prostate fibroblast cells also did not result in the oxidation of thiols (Figure 10C).

### 3.12. LNCaP Cells Have Twice as Much Catalase Activity as PC3 Cells and MnP Treatment Does Not Affect Catalase Activity

**Figure 11:**
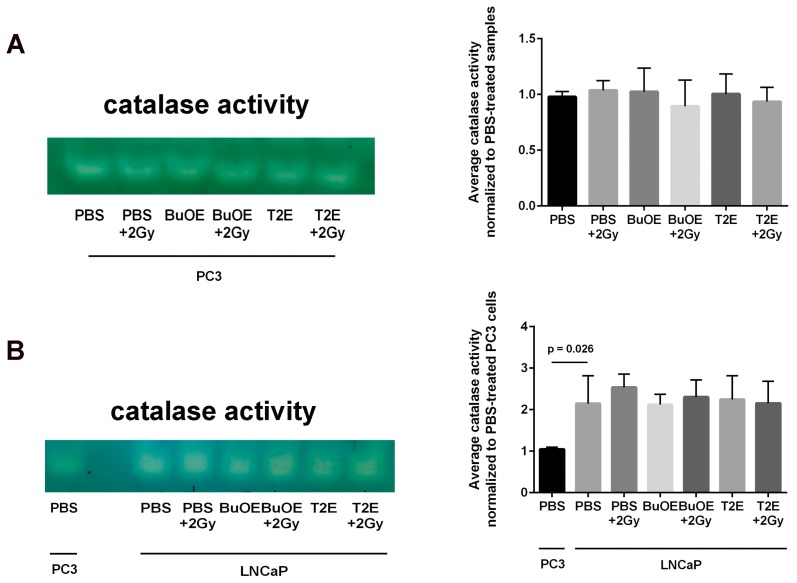
LNCaP cells have significantly more catalase activity than PC3 cells. (**A**) Left: A representative catalase activity gel for PC3 cells treated ± radiation and/or ± MnTE-2-PyP or ± MnTnBuOE-2-PyP. Right: Densitometric analysis of catalase activity gels; (**B**) Left: A representative catalase activity gel for LNCaP cells treated ± radiation and/or ± MnTE-2-PyP or ± MnTnBuOE-2-PyP. PC3 cells treated with PBS are also loaded on the same gel to compare the two cell types. Right: Densitometric analysis of catalase activity gels. The mean OD of the reactive bands in the control group was normalized to 1.0. Data represent mean ± SD from three independent experiments.

Because there was such a difference observed in hydrogen peroxide levels in PC3 cells as compared to LNCaP cells treated with MnPs, we investigated the baseline catalase activity in these two cell lines. LNCaP cells had significantly higher levels (2-fold greater) of catalase activity as compared to PC3 cells (Figure 11). Radiation alone or MnP treatment alone had no effect on catalase activity. The combination of radiation with MnPs also had no effect on catalase activity in either cell line (Figure 11).

## 4. Discussion

This study revealed that, in combination with radiation, MnPs decrease primary tumor size by inhibiting the proliferation of tumor cells in both LNCaP and PC3 orthotopic prostate tumor models. Thus, the overall median survival of the tumor bearing mice, treated with radiation and MnPs, was significantly increased. MnPs had no effect on metastatic progression, which is one of the major causes of prostate cancer related deaths. This study also demonstrated that MnPs protect tumor adjacent normal prostate tissue from radiation-induced lipid peroxidation. We also observed that the tumor redox environment affects the activity of the MnPs. In the oxidizing tumor environment, the MnPs do not scavenge superoxide, if anything there is a trend to increased amounts of superoxide levels in the prostate cancer cells. In contrast, in normal prostate cells we have previously shown that MnPs reduce superoxide levels in normal cells alone and in combination with radiation [12]. We speculate that the superoxide molecules autodismute into hydrogen peroxide. In LNCaP cells, there are two-fold higher levels of catalase activity as compared to PC3 cells. Thus, the hydrogen peroxide made in LNCaP cells when treated with MnPs is adequately scavenged and thiol oxidation is not observed in these cells. In contrast, PC3 cells cannot compensate for the increased levels of hydrogen peroxide with MnP treatment and the result is increased thiol oxidation. We only measured catalase activity; there could be differences in other peroxide removing enzymes as well between LNCaP and PC3 cells. This will be a future direction of these studies.

In combination with radiation therapy, or other chemotherapeutic agents, MnPs have been reported as anti-tumor agents. In a head and neck cancer model, MnTnBuOE-2-PyP sensitized tumor tissue to radiation [28]. In combination with other chemotherapeutic agents, MnTnBuOE-2-PyP acted as a pro-apoptotic molecule in glioblastoma multiforme [29]. Clonogenic survival of human pancreatic cancer cells was significantly decreased by MnP treatment in combination with ascorbate and gemcitabine [30]. We have reported previously that MnPs inhibit growth of prostate and colon cancer cells in combination with radiation or chemotherapies [10,11]. There have also been many studies that show MnTE-2-PyP or MnTnBuOE-2-PyP protect normal tissues from radiation damage in a variety of models [10,12]. However, no one has shown that the administration of MnPs protects normal tissues but not cancer tissues in the presence of radiation simultaneously.

We have observed that both MnPs do not scavenge superoxide, or are unable to scavenge superoxide at a rate equivalent to its production in cancer cells. In fact, there is a trend for a slight increase in superoxide levels in cancer cells treated with MnPs. Both MnPs have been reported to enhance oxidative stress in cancer cells [16,17,18,29,31]. It has been theorized that the SOD mimics act as pro-oxidants in oxidizing environments, such as cancer cells, because the Mn metal itself becomes oxidized. In the native SOD protein, the metal active site is surrounded by a large protein, which protects the metal from reacting with anything but superoxide. Thus, SOD enzymes generally do not act as pro-oxidants under any conditions. However, the SOD mimics are very small molecules and the manganese metal is able react with a variety of small oxidizing molecules. Therefore, instead of donating electrons to reduce free radicals (as is observed in normal cells and is normally thought as to how SOD mimics function), the SOD mimic instead steals electrons and in the process oxidizes these molecules. Thus, the SOD mimic acts a pro-oxidant when the surrounding environment oxidizes the metal. We postulate that the SOD mimics are behaving as such in the cancer cells.

Superoxide and hydrogen peroxide levels are key factors in determining how cancer cells will respond to therapeutic agents [32]. Superoxide can promote cell cycle progression and tumor vessel formation. Low levels of hydrogen peroxide leads to caspase dependent cell death, while, high levels of hydrogen peroxide can activate caspase independent apoptosis as well as necrotic cell death [33]. MnPs were proposed as therapeutic molecules against cancer cells because they can lower superoxide levels and potentially increase the hydrogen peroxide levels in cancer cells [32,34]. We found that in combination with radiation, MnTE-2-PyP or MnTnBuOE-2-PyP increases hydrogen peroxide levels in the PC3 cells [24]. In contrast, the MnPs do not increase hydrogen peroxide levels in LNCaP cells because LNCaP cells contain more of the hydrogen peroxide scavenging enzyme, catalase. Therefore, it is possible that in PC3 but not in LNCaP cells, MnPs can activate the hydrogen peroxide mediated mechanism of cell growth arrest.

It has previously been demonstrated that MnPs inhibit tumor growth in combination with ascorbate because MnPs oxidize ascorbate to peroxides, which increases steady state levels of hydrogen peroxide and hydroxyl anions [35] and leads to a caspase independent cancer cell death [16,36]. MnTE-2-PyP has also been reported to cause metabolic quiescence, which could account for the reduction in tumor cell growth observed in MnTE-2-PyP treated cancer cells [37]. We showed that MnTE-2-PyP and MnTnBuOE-2-PyP regulates thiol oxidation of redox sensitive proteins in PC3 cells. Presumably, some of these oxidized proteins could alter signaling molecules and potentially affect cell fate determination. Activity of cell cycle regulatory kinases and phosphatases are regulated by modulation of their oxidation state [38]. Thus, these proteins are potential targets of MnP-mediated cell growth arrest. One or any combination of these mechanisms could be considered as probable causes of MnP-mediated tumor growth arrest in PC3 models. Identification of the oxidized proteins in PC3 cells are planned for future experiments.

In our study, MnPs enhanced the anti-tumor effect of radiation. This data is also supported by another study showing that MnTE-2-PyP significantly controls tumor growth by decreasing the vascular density of the tumor [39]. Previously, C57BL/6 mice bearing RM-9 prostate tumors that were treated with MnTE-2-PyP showed reduced tumor growth due to activation of the immune system, specifically elevation of T helper, cytotoxic T cells and Natural Killer T cells [14]. Since we were using athymic mice to grow the human prostate cancer cells orthotopically, we did not observe these changes to the immune system. Therefore, we speculate that if we used a syngeneic mouse cancer model or a humanized mouse model, we may observe an increase in anti-tumor T cells and this may further inhibit the prostate cancer growth. These important studies need to be done to investigate the role of the immune system in MnP treatment of cancer in combination with radiation.

It has previously been reported that MnTE-2-PyP reduces the PCNA levels in a skin cancer model [40]. In our study, we also observed a reduction of PCNA by MnPs when combined with radiation in both tumor models. In replicating cells, PCNA acts as a harbor for the DNA replication-initiation protein machinery on the replication fork [41]. Depletion in PCNA causes a stall in DNA replication, which ultimately leads to an accumulation of cells in the S phase of the cell cycle [42]. Therefore, reduction of PCNA in both of the tumor types by MnP treatment and radiation, suggests probable growth arrest of tumor cells. This is likely another mechanism by which MnPs cause reduction of tumor size in combination with radiation.

There are vast differences in cell signaling and survival strategies in prostate cancer cells from early and late stages of prostate cancer. In the early stage, prostate tumor cells are confined to the prostate and the cancer cells are dependent on androgens for survival [43]. As the disease progresses, the tumor cells lose their dependency on external androgen for their survival [44,45,46]. Either, the cancer cells produce intracellular androgen for their survival or they utilize a ligand independent activation of the androgen receptor, which leads to activation of downstream survival mechanisms [47]. The androgen deprivation therapy fails at this point of disease progression. Therefore, the treatment protocols for early and late stages of prostate cancer vary differently. LNCaP cells are responsive to androgens and are not invasive. PC3 cells on the other hand are androgen independent and are highly metastatic. Therefore, LNCaP and PC3 cells can be considered representatives of early and late stages of prostate cancer, respectively. In our study, in combination with radiation, MnTE-2-PyP and MnTnBuOE-2-PyP inhibited tumor growth and protected the normal tissues in both prostate orthotopic models. Therefore, it can be concluded that in combination with radiation therapy, MnPs can inhibit both early non-metastatic and late-castration resistant prostate cancer growth.

In prostate cancer, the majority of patients receive radiation therapy. One of the major disadvantages of radiation is that it causes oxidative damage in the normal tissue adjacent to the tumor. Radiation enhances superoxide-mediated myofibroblast and senescent phenotypes in fibroblasts [48,49]. Radiotherapy for prostate cancer causes fibrosis and the dysfunction of the normal tissues in the radiation field and nearby tissues such as prostate, bladder, seminal vesicles, and bowel. Our study revealed that, MnP treatment during and after radiation, caused a significant reduction in radiation-mediated production of 4-HNE, a marker of lipid oxidation, in the tumor adjacent normal prostate glandular region. The MnTnBuOE-2-PyP treated animals were more protected from radiation damage than MnTE-2-PyP treated animals. This is probably due to the location of the MnPs inside the cells. MnTnBuOE-2-PyP is more lipophilic as compared to MnTE-2-PyP, so MnTnBuOE-2-PyP is likely in higher concentrations in the lipids than MnTE-2-PyP and, thus, is better able to protect from lipid oxidation [50].

There is a scarcity of effective radioprotectors for prostate cancer patients. Using antioxidants as radioprotectors has been controversial because it is feared that tumor tissue will be protected from radiation killing. In previous clinical trials, cancer patients receiving supplementation of β-carotene; or vitamins A, B, C, E, D3, or K3; or selenium; or cysteine and glutathione did not protect tumors with routine radiation or chemotherapy treatment [51]. However, there has been a lack of efficacy showing that vitamin supplementation protects normal tissues from radiation damage. This is likely due to these vitamins being poor ROS scavengers, not localizing to the area where free radicals are produced and were not given at high enough concentrations to be efficacious as free radical scavengers. Our study further illustrates that the redox active molecules, MnTE-2-PyP and MnTnBuOE-2-PyP, are not only effective radioprotectors, but also inhibit cancer growth in combination with radiation therapy. Thus, this study suggests that use of MnPs during and post-radiation in prostate cancer can significantly enhance the protection of normal tissues against side effects associated with prostate cancer therapy, while simultaneously inhibiting prostate cancer tumor growth. We believe that the addition of MnPs will result in better treatment of prostate cancer and improve the overall quality of life of patients undergoing radiation therapy for prostate cancer.
